# The Treatment of Neurasthenia1Read before the Medical Society of the County of Erie, June 8, 1894.

**Published:** 1894-10

**Authors:** William C. Krauss

**Affiliations:** Buffalo, N. Y.


### THE TREATMENT OF NEURASTHENIA.[1]

BY WILLIAM C. KRAUSS, M. D., Buffalo, N. Y.

WITH the few hours' notice that the writer had in which to prepare a paper for this society, it will be impossible to enter into a critical study, or even to enumerate the modes of treatment which have been devised by neurologists the world over for this common and omnipresent malady. I can only give you the methods of treatment which I pursue, and which, in the majority of cases—not every case—have given satisfaction and beneficial results. Some forms of neurasthenia are amenable to no form of satisfactory treatment, partly due to the patient and partly to the physi-

1. Read before the Medical Society of the County of Erie, June 8, 1894.

cian who has permitted his case to become chronic, almost petrified, before seeking the neurologist's aid.

It might not be amiss, before discussing the treatment of the patient, to look him over and see what symptoms he offers, and what right the specialist has to class him in this aristocratic circle of invalids. The idea is gaining ground that specialists must discover some symptoms, present or absent, to make his diagnosis and hold the case, and when no other form of disease can be consistently defended, then to class him, if he be a nervous case, among the neurasthenics or hysterical. This view is partly right, but for the most part wrong. Partly right, because the boundary line between *health* and neurasthenia is traced in the sand; wrong, because, although multiparous, the symptomatology of neurasthenia is quite distinct and well-defined from its neighbors, especially hysteria.

A neurasthenic individual is generally endowed with a certain quantity and quality of brain force, and in this respect he differs greatly from the hysterical, who possesses these qualities only in negative quantities. As a result of overwork, overstimulation, overindulgence, overworry, along with want of exercise, rest and enjoyment, he discovers, unless his wife has already told him, that he has become peevish, morose, inconsiderate, and a fault-finder of things in general. His mind, like his bowels, instead of being active has become constipated. Sleeplessness, forgetfulness and anxiousness, all conspire to make him that *bête noir* nervous, and he passes beyond the border line of health. A dull heavy ache at the occiput, sometimes at the vertex, and a corresponding heavy feeling at the lumbar region of the spine, are the only symptoms of pain that he can discover. Irregular and illy-defined symptoms, from every organ and tissue of the body, may now be expected, among the more important being palpitation of the heart, tachycardia, sinking spells, disorders of the vaso-motor system, as hot flushes, sense of heat, profuse perspiration of face and extremities; the stomach contributes such symptoms as loss of appetite, indigestion, ructus; the liver in many cases refuses to act because of insufficient nerve supply, giving rise to jaundice; the kidneys, from the same motives, yield urine sparingly and of high specific gravity; the genital organs are temporarily crippled; the muscular system is enfeebled, and even the soul itself sympathizes with the flesh and becomes indifferent and impassive.

One symptom especially, to which I would call your attention, is the irregularity of the pupils of the eyes. Text-books tell us that when the pupils are irregular, one contracted and one dilated, severe cerebral disturbances are present. I cannot subscribe to this teaching, as in all the cases of neurasthenia with vaso-motor disturbances that I have thus far observed, the pupils have been irregular, and they are so because the sympathetic nerve is implicated along with the others.

Another symptom which I have observed, more especially the past Winter and Spring, is an icterus, coming on very suddenly, lasting a few days, then, sometimes without hepatic stimulants, rapidly subsiding. This phenomenon is undoubtedly due to a weakened action of the hepatic branches of the vagus nerve, which, like the other nerves, is below par. I sometimes think that the vagus plays a very important rôle in the causation of neurasthenia, for certain it is that it becomes sorely afflicted in this disease.

This, then, is a very brief delineation of this affection, and to find the wherewithal to combat it, is the object of my presenting this paper today.

As regards treatment, I divide my cases into three classes: (1) those with a lithemic element; (2) those without a lithemic element; (3) those with marked vaso-motor disturbances.

In the three classes I ascertain the cause, or supposed cause, and insist upon its being removed, if possible. I then divide the first and second classes into asthenic and sthenic, and give the former about two ounces of hot black coffee before arising in the morning, followed in half an hour by a tepid sponge-bath for five minutes, then rapidly drying and dressing for breakfast. The sthenic plethoric cases receive one-half to one ounce of some saline cathartic, followed by a cold sponge bath and, if he has the conveniences, a cold plunge. A feeling of relief and pleasure is soon experienced by these sufferers and they await with delight their early morning bath. At night, a warm or hot general bath is taken just before retiring.

In all the classes, electricity is a prime factor in the treatment. The electrodes being placed at the neck and sacral region, a descending and ascending galvanic current of from 3 to 5 milliamperes is employed for from five to eight minutes. The positive pole is then applied to the forehead and a current of 1 to 1½ milliamperes is turned on for five minutes. The electrodes are then applied to the hands and a Faradic current is given, as strong as the patient can conveniently endure, for five minutes. The electrodes have 40 and 70 square centimeters surface respectively, thus giving a large contact and a steady current. This electric treatment is carried out three times weekly in the severe cases, once or twice weekly in the simple cases. In some of these I prescribe a course of massage along with regular exercise and amusements. In the lithemic cases I prescribe a tablet containing:

| | | |
|---|---|---|
| Lithium carbonate | 3 | grains. |
| Phosphate of iron | $\frac{1}{3}$ | grain. |
| Ext. nucis vomicæ | 1-9 | " |
| Arseniate of soda | 1-27 | " |

One to three tablets after meals. I have seen very good results follow the administration of this tablet, combining as it does an antilithemic with a nerve tonic treatment without giving rise to any gastric distress or instatia. Many of these patients cannot take iron in any form, either from imagination or fact, and refuse iron tablets with great persistency. The phosphate of iron, in this combination, produces no disagreeable effects and is well borne even by the most delicate and fastidious stomach. Instead of swamping these patients with manufactured or natural lithia waters, as is so often recommended, I advise drinking a glass of water after taking the tablets just referred to, and thus the use of lithia waters is dispensed with.

I have also used this tablet in cases of chronic rheumatism, and have had good reports from those taking them.

In the non-lithemic cases I prescribe the bromides and hyoscyamus with the spirits of lavender as a vehicle, and a nerve tonic containing the albuminate of iron, tincture nux vomica, phosphoric acid, with pepsin cordial as a vehicle.

Where there are special indications for treatment, as, for instance, insomnia, I give preference to trional in 5 and 10 grain doses. It is now over two years since I first began the use of this drug as an hypnotic, and, with but one or two exceptions, I have invariably had excellent results without any disagreeable after-effects. In those cases with obstinate gastric disorders, besides the treatment outlined above, I generally prescribe nux vomica before meals and dilute nitric or phosphoric acid after meals.

Constipation, if rebellious to the general treatment, yields readily to the aloin, strychnia, belladonna and cocoa pills, one to two pills before retiring.

Neurasthenics with vaso-motor disturbances have given me much annoyance and trouble, and I am far from satisfied with their progress under any kind of treatment. I have tried almost everything on these cases, and although getting some results with electricity and strophanthus, still they react grudgingly, and after weeks of treatment they are in nearly the same condition as before. The rest treatment acts quite well on some of these patients, but it is the exception and not the rule that they improve under it. Some of my patients, after an interval of months, tell me that outdoor life has accomplished as much as anything toward restoring them to health. Just why these cases are so intractable, I have no explanation to offer, and would be pleased to get some hints on this point in the discussion.

I have not attempted to enter into a serious study of this affection, but merely to outline my manner of treating these cases, with a hope of starting a discussion, which, after all, is the most important feature of any medical meeting.

